# Global Elimination of Lymphatic Filariasis: Addressing the Public Health Problem

**DOI:** 10.1371/journal.pntd.0000741

**Published:** 2010-06-29

**Authors:** David G. Addiss

**Affiliations:** Fetzer Institute, Kalamazoo, Michigan, United States of America; University of Kelaniya, Sri Lanka

## Background

On July 15, 1997, two months after the World Health Assembly (WHA) passed a resolution calling for the “global elimination of lymphatic filariasis (LF) as a public health problem,” a small group of public health leaders and scientists gathered at Magnetic Island, near Townsville, Australia. They were meeting to consider the elements of a program to achieve such a lofty goal. The evening before the meeting began I asked several of those present, all veterans of global efforts to eradicate smallpox, polio, or Guinea worm disease, whether the LF elimination program should concern itself with providing care to those who already suffer the clinical manifestations of LF. Their response was uniformly negative: LF elimination should focus solely on interrupting transmission of the parasite. As with other disease eradication efforts, the intended beneficiaries were future generations; saddling the LF elimination program with responsibilities for clinical care could dilute focus, divert resources, and complicate strategies and partnerships.

Two days later, the group unanimously endorsed a “two-pillar” strategy that included both interrupting transmission and providing care for those with disease [1]. Several arguments had shifted the group's position. First, there was the ethical issue: how could one ignore the suffering of 15 million people with lymphedema and 25 million men with urogenital disease, principally hydrocele? Hydrocele is readily treated with surgery, and evidence was beginning to accumulate that simple measures, including hygiene and skin care, could help arrest the progression of lymphedema [2], [3]. Second, the “public health problem” to which the WHA resolution referred was clinical disease; by itself, the presence of microfilaria in the blood does not constitute a public health problem. In affected communities, clearing the blood of microfilaria through annual mass drug administration (MDA) can interrupt transmission of the parasite. However, the scientific evidence remained divided on what effect, if any, these drugs have on established disease [4]. Finally, and perhaps most importantly, it was thought that providing care for those with filariasis-associated morbidity could increase community acceptance of MDA.

The World Health Organization (WHO) launched the Global Programme to Eliminate Lymphatic Filariasis (GPELF) in 2000. Ten years on, progress in scaling up MDA has been phenomenal; 496 million persons received antifilarial drugs in 2008 [5], yielding impressive global health benefits [6]. In contrast, despite excellent pilot programs (e.g., [7]–[9]) and some at the state and national levels [10], morbidity management has generally languished.

What are the reasons for this imbalance? For one, the concerns expressed at Magnetic Island had merit. The single focus of other disease elimination programs enabled them to be streamlined and efficient. The dual goals of interrupting transmission and managing morbidity may require different approaches, skills, and timeframes. Given limited resources and the ambitious goal of interrupting transmission by 2020, MDA has taken priority. Despite the experience of those who advocated a “two-pillar” strategy, there has been no scientific evidence that lymphedema management actually improves acceptance of MDA. Thus, it has been difficult to dispel the notion that investing in lymphedema management drains limited resources from the primary goal of interrupting transmission.

In this issue of *PLoS Neglected Tropical Diseases*, Paul Cantey and colleagues elegantly demonstrate how program evaluation, at its best, can both improve program effectiveness and contribute to scientific knowledge. They provide the first solid evidence that lymphedema management, far from competing with MDA, actually enhances drug coverage [11]. The importance of high coverage for interrupting LF transmission cannot be overstated; indeed, success depends on it [12].

## Findings

Following the 2008 MDA in Orissa, India, an area with substantial morbidity, Cantey and colleagues used a well-accepted cluster survey design to assess drug coverage and identify barriers to compliance. The survey was meticulously conducted in three areas where diethylcarbamazine (DEC) had been distributed 6–8 weeks previously. Residents of one area received the standard pre-MDA education that is typical for Orissa (MDA-only). Another area had enhanced community-based pre-MDA education that was designed to address barriers to compliance identified in the 2007 MDA (Com-MDA) [13]. In the third area, which also received enhanced community-based pre-MDA education, a lymphedema management program had been initiated earlier in the year (Com-MDA+LM). Patients and their families were trained in basic lymphedema self-care and, interestingly, the public also was educated as to the benefits of these measures for affected persons.

The results were striking. In an area where compliance with MDA chronically hovered around 50%, the proportion of survey respondents who reported taking DEC was 52.9%, 75.0%, and 90.2%, respectively. Compliance was significantly higher in the Com-MDA+LM area than in the Com-MDA area—and well above the threshold considered necessary to interrupt transmission [11]. Further, at the individual level, knowledge of at least one component of lymphedema self-care was significantly and independently associated with adherence to DEC. This association was observed even among those who did not have a family member with leg swelling.

## Implications

These provocative and encouraging findings raise important questions for the GPELF and for further research. It is notable that the work was done in India, the country with the greatest LF burden. Can the results be replicated in other settings, in areas with less intense morbidity or different economic conditions? To what extent did they depend on a solid public–private partnership that utilized the ample community connections and mobilization skills of the private partner, the Church's Auxiliary for Social Action? What specific components of the lymphedema management program had the greatest effect on drug coverage, and why? The results both highlight the importance of lymphedema care and suggest that community awareness of its availability and benefits may be the key to increasing acceptance of MDA.

## Future Directions

Scientific evidence that lymphedema management can enhance MDA coverage and thereby hasten the interruption of LF transmission comes at a critical juncture in the life of the GPELF. In some countries, a boost in drug coverage provided by lymphedema management could mean the crucial difference between success and failure. This approach may be especially effective in areas where systematic noncompliance with MDA has been identified as a potential barrier to LF elimination [14].

In other countries, LF transmission appears to be on the verge of elimination, and Ministries of Health will soon be seeking official verification of this accomplishment. The imprecise wording of the WHA resolution in 1997 served well for mobilizing a variety of partners with different interpretations of the final endpoint, ranging from reduced transmission to global extinction of the parasite. Now, however, precise verification criteria are needed. It would be inconsistent with the WHA resolution to verify “elimination as a public health problem” solely on the basis of infection.

The findings of Cantey et al. will stimulate a fresh and vigorous discussion regarding the relationship between the dual programmatic goals of interrupting transmission and reducing current LF-related suffering. They point the way toward a more comprehensive LF program that is integrated into the health system [15] and more fully equipped to truly eliminate LF as a public health problem.
